# An Outbreak of Varicella among Schoolchildren in Taipei

**DOI:** 10.1186/1471-2458-11-226

**Published:** 2011-04-12

**Authors:** Chao-Chih Lai, Szu-Ching Chen, Donald Dah-Shyong Jiang

**Affiliations:** 1 Emergency Department of Taipei City Hospital, Ren-Ai Branch, Taiwan, R.O.C; 2 Health Center of an Elementary School in Taipei, Taiwan, R.O.C; 3 FETP, Centers for Disease Control, Taiwan, R.O.C

## Abstract

**Background:**

The reported cases with varicella have not decreased and outbreaks of varicella among vaccinated children continue to be reported 9 years after the public vaccination program in Taipei. We investigated an outbreak to determine varicella vaccine coverage and effectiveness.

**Methods:**

An outbreak occurred in an elementary school which located in southern Taipei from April 2007 through May 2007. A retrospect cohort study was performed by using a self-administered questionnaire for parents.

**Results:**

Ten out of sixteen varicella cases were vaccinated. Overall vaccine coverage was 71.2%. The common reasons for not receiving varicella vaccine were that varicella vaccine was unavailable because the student didn't live in Taipei (29.4%) or the children could not be vaccinated due to certain illnesses (23.5%). The sensitivity and specificity of self-reported vaccination status was 0.900 (95% CI: 0.864, 0.935) and 0.611 (95% CI: 0.514, 0.701).

The vaccine effectiveness was 69.3%-100.0% against any disease severity of varicella. Overall vaccine effectiveness against moderate or severe varicella was 85.5%. Attending cram school was associated with the risk of developing the varicella illness (RR: 13.39; 95% CI: 5.38, 33.31). Unvaccinated students tended to show moderate to severe (>50 lesions) afflictions of the disease (RR: 4.17; 95% CI: 1.15, 15.14).

**Conclusions:**

Because of the low vaccination coverage, varicella outbreaks continue to be reported in Taipei. Increasing vaccine coverage and second dose vaccination for increasing vaccine effectiveness may be considered.

## Background

**Figure 1:**
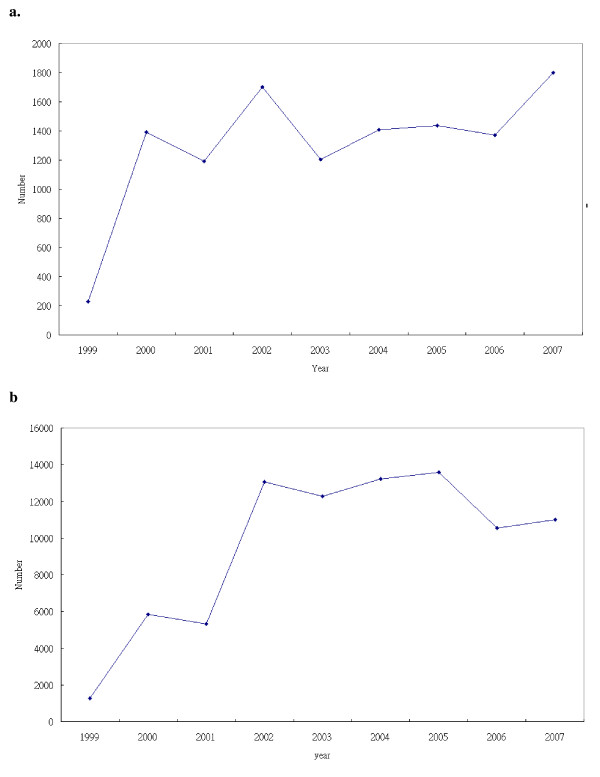
**The yearly reported cases with varicella in Taipei (A) and Taiwan (B), 1999-2007**. (A) The yearly reported cases with varicella in Taipei, 1999-2007. (B) The yearly reported cases with varicella in Taiwan, 1999-2007.

A live attenuated varicella vaccine was made commercially available in Taiwan in September 1997. It has been included in public vaccination programs in Taipei city and Taichung city/county since 1998 and 1999 respectively [1]. It could be provided by self-paid option in other areas of Taiwan. Varicella disease has been included in the reports of National Notifiable Disease Surveillance System in Taiwan since 1999. A routine varicella vaccination for all children born after 2003 and aged 12 months or older was implemented in Taiwan since 2004. Varicella disease has declined dramatically 5 years after the introduction of vaccine in the United States [2]. Although some studies show complications and significantly declined hospitalization rates after vaccine introduction in Taiwan [3,4], the reported cases with varicella has not decreased and outbreaks of varicella among vaccinated children continue to be reported in Taipei and Taiwan [5,6].(Figure 1a, b)

The vaccine effectiveness ranged from 44 to 100 percent against varicella disease of any severity [7-14]. But it hasn't been studied in Taiwan. Vaccine effectiveness could be potentially affected by the duration of immunity, the optimum age for vaccination, and other factors. Some studies have suggested that immunity may wane after vaccination [13,15,16].

Varicella outbreaks in several elementary schools were detected in Taipei in 2007. It remains unclear whether low vaccine efficacy or low vaccine coverage resulted in outbreaks in elementary schools in Taipei. We investigated a varicella outbreak of 16 cases which, occurred on April 4, 2007, in an elementary school to determine vaccine coverage and effectiveness, and to compare disease severity among vaccinated and unvaccinated students.

## Methods

From April 2007 through May 2007, a varicella outbreak occurred in an elementary school located in a southern district of Taipei. There were 1,038 students attending grades 1-6 in the school. All classes with afflicted cases were situated in the same building. All 16 cases confirmed by the physician were suspended from classes and stayed home for either 5 or 7 days. A retrospective cohort study recruiting students of the involved grades (1, 3, and 4) was performed by sending a self-administered questionnaire to their parents during this outbreak and finished after summer break in September. This study was approved by the Taipei City Hospital Institutional Review Board.

### Questionnaires

The contents of the questionnaire (see additional file 1) included demographic information, vaccination history, prior chickenpox history, conditions associated with varicella disease including the severity, complications, hospitalization, duration of rashes, and household transmission. The vaccination history of students and the date of receiving the vaccine were verified by vaccination records.

### Case definition

A case of varicella was defined as an acute maculopapulovesicular rash without other explanation occurring in a student without a prior history of chickenpox from April 4, 2007 through May 18, 2007. Diagnosis was made on the basis of physician. Illness was classified as mild (<50 lesions), moderate (50-500 lesions), or severe (> 500 lesions). Students without history of varicella were classified as vaccinated or unvaccinated. Students with prior history of varicella were excluded from the analysis of vaccine effectiveness.

### Statistical Analysis

The data were entered into Epi Info (version 3.43; Centers for Disease Control and Prevention) and analyzed with the SAS software (release 9.1, SAS institute). The validity of self-report compared with vaccination records was assessed by calculating rates of sensitivity and specificity, assuming the vaccination records to be the "gold standard" of accuracy. Positive Predictive Value (PPV), the complement of the false positive rate, and Negative Predictive Value (NPV), the complement of the false negative value (FNV), were also calculated. Kappa statistics were also calculated to determine the degree of agreement. Relative risks (RR) and 95% confidence intervals (CI) were calculated, with CI excluding 1.0 considered statistically significant. Fisher's exact test was used for the comparison of proportions and 2 sided *p *values were reported, with significance level of α = .05. Medians were compared by using Wilcoxon rank-sum test. Vaccine effectiveness rate was calculated by cohort method [17]. To calculate the vaccine effectiveness as [(ARU-ARV)/ARU] × 100, which ARU means the attack rate in the unvaccinated population and ARV means the attack rate in the vaccinated population.

## Results

Among the 522 students which attended grades 1, 3, and 4 in the elementary school, there were 510 (97.7%) questionnaires returned. Seven students were transferred to another school during the period of investigation and 5 parents didn't response to the questionnaire. Based on vaccine records, there were 321 (71.2%) vaccinated students and the vaccine coverage of grade 1, 3, 4 were 89.1%, 70.6%, and 45.1% respectively. The mean age of study population was 9.1 (± 1.4) years old. Characteristics of students who were enrolled in the investigation are summarized in Table 1. A total of 227 students attended cram school after classes.

**Table 1:** Characteristics of 510 Students who attended grades 1, 3, and 4 in one elementary school during the varicella outbreak period, from April 2007 to May 2007.

Characteristic	Grade 1 (N = 155)		Grade 3 (N = 160)		Grade 4 (N = 195)	
	**Students with varicella**	**Students without varicella**	**Students with varicella**	**Students without varicella**	**Students with varicella**	**Students without varicella**

Sex						
Female	5 (3.2)	77 (49.7)	0 (0.0)	76 (47.5)	1 (0.5)	90 (46.2)
Male	8 (5.2)	65 (41.9)	2 (1.3)	82 (51.3)	0 (0.0)	104 (53.3)
Susceptibility						
History of varicella	0 (0.0)	17 (11.0)	0 (0.0)	28 (17.5)	0 (0.0)	73 (37.4)
Unvaccinated	4 (2.6)	11 (7.1)	1 (0.6)	30 (18.8)	1 (0.5)	66 (3.0)
Vaccinated	9 (5.8)	114 (73.5)	1 (0.6)	100 (62.5)	0 (0.0)	55 (28.2)

Vaccine coverage	134 (89.1)		115 (70.6)		72 (45.1)	

**NOTE**: Data are of no.(%) of students.

Vaccine coverage = [The Number of vaccinated students]/[The Number of total students]

The sensitivity of self-reported vaccination status was 0.900 (95% CI: 0.864, 0.935) and the specificity of self-reported vaccination status was 0.611 (95% CI: 0.514, 0.701). The kappa statistics was 0.324 (95% CI: 0.221, 0.427). Positive Predictive Value (PPV), the complement of the false positive rate, was 0.784 (95% CI: 0.739, 0.829). Negative Predictive Value (NPV), the complement of the false negative value (FNV) was 0.611 (95% CI: 0.499, 0.724). No significant difference was found in different sex or age. Overall vaccine coverage was 81.6% according to the self-reported vaccination status.

There were 101 (19.8%) unvaccinated students in the study group. The reasons for not receiving varicella vaccine were as following: (1) varicella vaccine was unavailable because the student didn't live in Taipei (29.4%); (2) the children can not be vaccinated due to certain illnesses (23.5%); (3) had varicella before vaccination (19.6%); (4) varicella is not a severe disease (9.8%); (5) the safety of new vaccine is uncertain (5.9%); (6) no information about varicella vaccine was told (5.9%); and (7) not protected by vaccine completely (3.9%).

**Figure 2:**
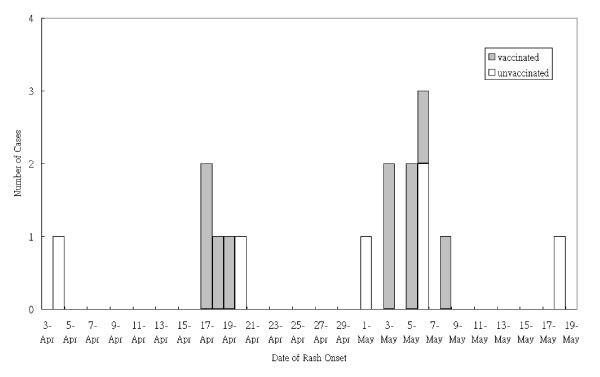
**Cases of varicella in one Taipei Elementary School between April 2007 and May 2007, according to the vaccination status of the children and date of rash onset**.

Sixteen cases were associated with the varicella outbreak which began in April 4, 2007 and continued until May 18, 2007 (Figure 2). The mean age of cases was 8.4 (± 1.7) years old, and 11 of these cases (61.1%) were boys. There were no cases in students with history of varicella disease. No students with varicella came from the same household. Of these cases, there were 10 vaccinated students with varicella. A total of 6 classes had varicella cases in the elementary school, 3 classes were grade 1 and 2 classes were grade 3 and one class was grade 4. Ten of the cases attended cram schools after classes. The index case was in a susceptible unvaccinated first-grade with known expose to the other student with varicella from another school while attending cram school after classes. Attending cram school after classes was associated with the risk of developing varicella illness (RR: 13.39; 95% CI: 5.38, 33.31) in this outbreak.

Among those without a history of varicella in grade 1, there were 4 cases out of the 15 unvaccinated students (ARU: 26.7%) and 9 out of the 123 vaccinated students (ARV: 7.3%). Therefore, vaccine effectiveness was 72.6% for any varicella illness in grade 1. It follows that vaccine effectiveness was 69.3% in grade 3 and 100.0% in grade 4. Overall vaccine effectiveness against moderate or severe varicella was 85.5%.

Of the 16 cases in the outbreak, 10 (62.5%) cases occurred in vaccinated students. There were 5 (83.3%) cases with at least 50 lesions in unvaccinated students with varicella disease, compared with 2 (20.0%) cases with at least 50 lesions among vaccinated students (RR: 4.17; 95% CI: 1.15, 15.14). The median duration of the illness in vaccinated students was 6.0 days (range: 2~10days), compared with 8.5 days (range: 6~15days) among unvaccinated students (*p *= 0.370). No child required hospitalization.

## Discussion

To our knowledge, this is first study of varicella outbreaks since the implementation of the vaccination program in Taiwan. The overall vaccine effectiveness in this investigation was the same as previous reported in most studies [7-14]. Vaccinated students with varicella had fewer lesions.

In the validation study of self reported varicella vaccination status, we found that over-reporting (1-specificity) was common. Age and sex did not impact significantly on validity estimates. Self-reported varicella vaccination coverage among the school children over-estimates true vaccination coverage by about 10% (81.6% versus 71.2%). Although sensitivity of self-reported influenza vaccination status was high (90.0%), specificity was poor (61.0%).

We found that attending cram school after classes, due to its crowed space, was a risk factor for developing varicella illness in Taipei. The crowded space increases the contact rate between children. Also, the index case was exposed to varicella in cram school A. Therefore, attending cram school resulted in not only the spread of varicella disease between schools or classes but also developing varicella illness in this outbreak. Therefore, the control and investigation of school outbreaks should include the private cram schools in Taipei.

The number of cases with varicella disease hasn't declined in National Notifiable Disease Surveillance System 9 years after introduction of vaccine in Taipei, because many unvaccinated students moved in Taipei city from other county which resulted in low vaccine coverage rate.

We believe that vaccine coverage rate in Taipei was elevated after varicella vaccine implemented in all Taiwan since 2004. Though the vaccine coverage reached 89.1% in first grade students and 92% in some class of grade 1, school outbreak couldn't be prevented in Taipei. Modeling predicts that 97% of varicella vaccine coverage rate will need to be reached to prevent outbreaks [18]. Therefore, we conclude that school varicella outbreaks that occur in Taipei result from low vaccine coverage. But other risk factors, such as high contagiousness of varicella, breakthrough infection, un-vaccined children moving into Taipei, could be present, further study should be done in the future.

After 8 years of implementing varicella vaccination program in Taipei, varicella incidence hasn't declined dramatically and outbreaks continue to be reported. Low vaccine coverage, which was attributed to lack of nationwide varicella vaccination program can't prevent the occurrence of outbreaks. In addition, we should solve the problems of sick children without vaccination and parents' misconception about vaccination in order to promote high vaccination coverage. However, even with high vaccination coverage, one dose varicella vaccination did not provide sufficient herd immunity levels to prevent community transmission [19,20]. A second dose of varicella vaccine could increase vaccine effectiveness and decrease breakthrough rate [21]. A 2-dose varicella vaccine schedule has been recommended for children in United States since 2006 [19]. However, one study showed the vaccine effectiveness of 1 and 2 doses were similar [22]. Second dose vaccination program to prevent community transmission may be considered in Taiwan.

## Conclusions

Attending cram school after classes was the risk factor in this outbreak. Because of the low vaccination coverage, varicella outbreaks continue to be reported in Taipei. Expanding vaccine coverage and second dose vaccination for increasing vaccine effectiveness may be considered.

## Competing interests

The authors declare that they have no competing interests.

## Authors' contributions

DDSJ conceived the study, collaborated on the study design, the structuring of the statistical analysis, interpretation of the data, and writing of the manuscript. CCL collaborated on the design of the study, was responsible for overall conduct of the study, collaborated on the analysis and interpretation of the data, and took the lead in drafting the manuscript. SCC collaborated on the design of the study, was responsible for data collection and data management, and participated in the writing of the manuscript. All authors read and approved the final manuscript.

## Pre-publication history

The pre-publication history for this paper can be accessed here:

http://www.biomedcentral.com/1471-2458/11/226/prepub

## Supplementary Material

Additional file 1 **Questionnaire**. Explanation of the structure and content of the questionnaire. Click here for file
