# Supplementary material for: An Outbreak of Varicella among Schoolchildren in Taipei
Source: BMC Public Health. 2011 Apr 12;11:226. doi: 10.1186/1471-2458-11-226 (PMC3095558; doi:10.1186/1471-2458-11-226)
Supplement: Additional file 1 — Questionnaire. Explanation of the structure and content of the questionnaire. [file 1471-2458-11-226-S1.PDF]

Varicella Investigation Questionnaire
For the Hsinlong Elementary School

Name: ________________

Grade: _______ Class: _______ Seat No.: _______

1. Has your child ever contracted Varicella?
   - ☐ Yes, this year.
   - ☐ Yes, before this year, approximately :____/___/__ (date).
   - ☐ Never.

2.Has your child ever received vaccine for Varicella?
   - ☐ Yes.
   - ☐ Unsure.
   - ☐ **No. If your child has not received vaccine for Varicella, please specify the reason:**
     - ☐ your child was ill at the time for receiving the vaccine
     - ☐ The household was not registered in Taipei City at the time, thus unable to receive free vaccination.
     - ☐ Because it is a new vaccine, possible side effects are worrying.
     - ☐ Contracting varicella is not serious, thus there is no need to receive the vaccine.
     - ☐ Other: ______________________________________

【If your child has never contracted Varicella, skip this part】

3. If your child has contracted Varicella before, how long did the rash persist?
   _____________ days

4. If your child has contracted Varicella before, approximately how many pockmarks appeared?
   - ☐ less than 50 lesions ☐ 50～249 lesions
   - ☐ 250～500 lesions ☐ more than 500 lesions

5. If your child has contracted Varicella before, was he/she hospitalized for it?
   ☐ Yes ☐ No

6. If your child has contracted Varicella before, was there fever?
   ☐ Yes ☐ No

7. If your child has contracted Varicella before, were there any serious complications (such as Pneumonia or encephalitis) that occured?
   - ☐ Yes, the complication was: ___________________ .
   - ☐ No.

8. When your child contracted Vericella, were there any other family members who also contracted the disease?
   - ☐ Yes. / How many persons were contracted? ______ persons. / Who were they?
     ____
   - ☐ No.
